# In memoriam: Charis Eng MD PhD (1962–2024)

**DOI:** 10.1530/ERC-24-0282

**Published:** 2024-12-18

**Authors:** Deborah J Marsh, Lois M Mulligan, Joanne Ngeow, Matthew D Ringel, Constantine A Stratakis

**Matthew Ringel** On behalf of the entire editorial team of *Endocrine-Related Cancer*, and personally as trainees and collaborators who at present serve as associate or senior editors of the journal, it is with profound sadness that we write this memorial to Prof. Charis Eng MD PhD, who passed away 13 August 2024. Prof. Eng served as Editor-in-Chief of *Endocrine-Related Cancer* from 2011 to 2021 and was dedicated to the journal before, during and after she served in that role. She had a remarkable impact on *Endocrine-Related Cancer*, moving the journal forward with great vision and energy while maintaining the strongest commitment to publishing the highest-quality original research and reviews, applying fair, ethical and rigorous peer-review processes. Charis was a world-recognized leader in cancer genomics and clinical genetics medicine, who enabled growth in *Endocrine-Related Cancer* in these and other areas critical for advancement of research and clinical care of endocrine cancers. She will be remembered by all of us as a superb and consequential researcher, editor, physician, leader, teacher, mentor, colleague and friend.

Charis served, since 2005, as the founding Director of the Genomic Medicine Institute at the Cleveland Clinic, where she held the Sondra J. and Stephen R. Hardis Endowed Chair. She was also a professor and vice-chair of the Department of Genetics and Genome Sciences at Case Western Reserve University College of Medicine. Over the course of her remarkable career, she made seminal observations regarding the cause and mechanism of genetic disorders, including the first identification of inherited *PTEN* mutations in Cowden syndrome, Bannayan–Riley–Ruvalcaba syndrome and autism, and published high-impact work defining the mechanisms and clinical management of patients with inherited *RET* mutations and multiple endocrine neoplasia type 2. She was a leader in defining the spectrum of *PTEN* hamartoma syndromes, leading the work in the clinical characterization of the syndrome and also in identifying factors that influence the variation in clinical presentations. Her work was highly collaborative, was international in scope, and was always inclusive of trainees and patients.

Prior to her time at the Cleveland Clinic, she served as the Director of the Division of Human Genetics at the Ohio State University, where she was faculty from 1999 to 2005, was promoted to professor, and held the Klotz Endowed Chair. It is in this context that we began our collaborations. Charis began her academic faculty career at the Dana–Farber Cancer Institute after completing her residency in Internal Medicine at Beth Israel and fellowships at the Dana–Farber, the University of Cambridge and the Royal Marsden NHS Foundation Trust in the UK. Over the course of her training and career, Charis published more than 500 peer-reviewed manuscripts, many in very high-impact journals, which are highly cited and have had a tremendous impact. She was recognized with numerous accolades for her research, clinical and mentoring excellence, including election to the National Academy of Medicine (USA), the American Society of Clinical Investigation and the Association of American Physicians, and fellowships in the National Academy of Medicine, the American Society of Clinical Investigation, the Association of American Physicians and the American Association for the Advancement of Sciences. She received research awards from the American Association for Cancer Research, the American Thyroid Association, the Endocrine Society and the American Cancer Society. She also received awards for excellence in mentorship from the American Medical Women’s Association. Charis will be missed deeply by all of us at *Endocrine-Related Cancer*, by those of us whose careers were impacted by her and those who benefited from the knowledge gained in her research to improve clinical practice.

**Lois Mulligan** Charis Eng and I came to know each other as post-doctoral fellows in the laboratory of Prof. Sir Bruce Ponder in the Cancer Research Campaign Cancer Genetics Research Group at the University of Cambridge. She joined the Ponder group just as we had identified mutations of the *RET* receptor in multiple endocrine neoplasia type 2 (*MEN2*) and became part of the team focused on driving that project forward. The laboratory was a heterogeneous multinational community, with diverse backgrounds and perspectives united in our shared research goals. This was an exciting time as we teased apart the *MEN2* disease mechanism, and explored the implications of the novel and unexpected discovery of a mutant oncogene that caused an inherited cancer syndrome. Charis brought her work ethic, and clinical and research background to the identification of *RET* mutations in *MEN2B* and familial medullary thyroid cancer. We worked together with Bruce Ponder in establishing the International RET Mutation Consortium, which brought together the worldwide experience of *MEN2 RET* mutations, and identified the earliest correlations of *RET* genotype with disease phenotypes that helped guide diagnosis and disease management. Together, this work confirmed for Charis how much can be accomplished by an integrated and committed team, and highlighted the impact of strong collaborative networks in tackling difficult clinical challenges that can change the conversation for patients with inherited cancers. Charis never looked back. She spent her career building consortia, initiating collaborations, nurturing her research team and always channelled efforts towards bettering the lives of cancer patients.

Charis Eng was a force to be reckoned with. Even as a post-doctoral fellow, she knew what she wanted to achieve. Importantly, she reached those heights by lifting up her friends, colleagues and trainees as she climbed. She leaves a broad network of past trainees, colleagues and patients who feel her passing. I will always remember her passion for her work, for good food, wine and conversation at her table, and her inimitable laugh. She lived her best life helping others to live theirs.

**Deborah Marsh** Charis had the vision to lead international consortia from very early in her career, and it was in this context that I first met her, at the Fifth International Workshop on Multiple Endocrine Neoplasia, Stockholm, Sweden, in 1994. Alongside Lois Mulligan and supported by Prof. Sir Bruce Ponder, she convened the International RET Mutation Consortium, delivering major insights into multiple endocrine neoplasia type 2. As a then Australian PhD student, I was warmly welcomed into this world of international science. Two years later, I joined Charis’ laboratory at the Dana–Farber Cancer Institute in Boston as her first post-doc. Leading teams around the world, Charis had used linkage analysis to map the location of the gene for the familial cancer disorder Cowden syndrome to chromosome 10q22–23. A collaboration with Ramon Parsons at Columbia University revealed *PTEN* as the gene that Charis had been tracking, and she found that germline mutations were causative for Cowden syndrome and the overgrowth disorder Bannayan–Riley–Ruvalcaba. Reporting this in two *Nature Genetics* papers in 1997, Charis was there at the beginning of our understanding of the tumour suppressor *PTEN*, now recognized as one of the most frequently mutated genes in human malignancy. Her name will always be synonymous with the *PTEN* field, a field in which she continued to make major discoveries until her passing.

As a post-doc, I worked hard alongside Charis, because when you train with Charis, you make the most of opportunities. Her laboratory grew with post-docs from Brazil, Germany, China and Switzerland in the 3 years that I shared this academic home. Her catchphrase when she arrived at the laboratory morning and afternoon was always ‘*What’s new and exciting!?*’, and she would rub her hands together in anticipation. Charis’ warmth and generosity were evident during this time. As trainees, we were frequently invited to events with visiting scientists and clinician-scientists, and there were many. For anyone who has ever dined with Charis, you would know that good food, conversation and wine flowed freely. Happily, these events, and her guidance of my own career, extended past my time as her post-doc, where I could always rely on her wise counsel up until very recently. Charis’ trainees, now located all over the world, are part of her legacy as we all step up to be the best that we can be in our chosen fields and honour her memory. I am sure that I speak for many in saying that Charis will be very much missed.

**Constantine A Stratakis** Charis and I met in 1995 as I was starting my laboratory, a small unit at the time in the Developmental Endocrinology Branch, NICHD, NIH, in Bethesda, MD, USA. We immediately felt that we had a lot in common, not only in terms of who we were and what we did, but also in our way of thinking. We, therefore, ‘clicked’: both foreigners, newly coined independent investigators in the very competitive environment of gene hunting for monogenic diseases in the mid-1990s; we joined forces and shared data, connections and networking. There were times in which we were talking daily about various issues.

I admired Charis! Open-minded, hard-working, honest and creative, she was the perfect person to consult with on academic matters, research ideas, new genes and their function. Charis was always able to give you another perspective on everything; her amazing foresight added another dimension on any discussion. She was warm and engaging, passionate about the things she cared for, fiercely supportive of not only her trainees but also her friends and collaborators: once you became part of this group, you knew that Charis would always be there for you. I shared with Charis a passion for wine; naturally, our dinners together were exercises in oenology and they are memorable for that reason.

Charis was, of course, an accomplished geneticist and researcher, and we talk a lot here about her work on *PTEN* and other diseases. However, as Matt pointed out above, Charis was also an accomplished executive leader: wherever she led (the Ohio State, the Cleveland Clinic and major Editor-in-Chief positions), she created programs, established laboratories, trained people, promoted faculty and other researchers, worked crazy hours, was passionate, and always with great integrity and vision. When I became Scientific Director at NICHD, NIH, I talked to her frequently for her advice. Once again, she showed her many attributes and magnanimity: her dependable, cool-headed advice would come my way whenever I needed her. In addition, we both went through difficult times as leaders: Charis was there for me (and I was there for her).

I miss Charis: I miss her devotion to our science, her tactful and diligent approach to academic issues, her loyalty to all of us, her friends and collaborators, her determination to do things the right way, her decisiveness and her advice. Our field misses a scientist and a leader who served with an unwavering devotion to discovery, building, training and mentoring.

**Joanne Ngeow** I first met Charis in 2008 when she was invited as the Ministry of Health Healthcare Manpower Development Plan Visiting Expert to Singapore. She also had a very interesting interview with the Singapore Medical Association that same year (https://www.sma.org.sg/news/2008/October-1/interview-with-professor-charis-eng). I remember our dim sum lunch at Shangri-La during her visit, like it was yesterday. I had already accepted a phase I clinical trial fellowship in Toronto and had not known that my lunch was an interview that my head of department had arranged for me with Charis. We had a very enjoyable afternoon that day, the first of many such meals over the years. A few days later, I was called in to speak with my head of department, who said that Charis had been very pleased with our meeting and that I should consider doing my fellowship in Cleveland in genomic medicine, as it was an area of great unmet need in Asia.

I trusted his advice and, like a good Singaporean, I did as I was told. The fellowship was a unique blend of bench and bedside training in translational cancer genomics and ahead of its time. I struggled with an initial lack of foundation in clinical genetics. I dearly miss her five to six pages of single-spaced annual appraisals, where she would highlight specific areas I should work on. I was initially very daunted, having come from an education system where such frank appraisals were rare, and it was indeed very hard to hear such harsh feedback. I am grateful though that I did not pack my bags to return home that first six very tough months. Somewhere in the mix of tears and frustration, I recognized that here was someone who really cared about my learning, and how rare and precious that was. However, I was not an exception: Charis took the time and care for such detailed appraisals for EVERY single member of her laboratory and her institute. Instead of one year, I stayed for four, because that was what it took for me, a novice in genetics, to be properly trained in the field. This gave me the time to learn, not only about the science, but also all the other skills for leading a team. I loved to watch Charis at a collaborator’s meeting: she was always so sharp and contributed her ideas freely. She was always also looking to add value to the team, and this is why she was such a trusted voice in so many research and guideline committees.

I explain this difficult start that I had with Charis because her successes were frequently remarkable: she often chose to mentor, not the brightest and best in the field, but those whom she could most add value to. Many diamonds in the rough. Women and trainees from very diverse backgrounds across the globe have all, likewise, benefited from mentorship in her laboratory and remotely. She only cared that you were curious and wanted to learn. She had little patience with ‘not serious’ trainees, but her generosity had no bounds for anyone who was committed to the work. She was a brilliant writer. I thoroughly enjoyed the many editorials we wrote together, which not only honed my writing skills but helped me gain confidence that my voice mattered, even as a young person in the field. I started a similar Cancer Genomic Medicine Fellowship at the National Cancer Centre Singapore, modelled very much after my own fellowship programme. We have now trained four clinicians in cancer genomic medicine from Southeast Asia, and we hope to continue Charis’ legacy through the future fellows that we train up. Charis taught me what it means to be an excellent clinical cancer geneticist, diagnostician, collaborator, science citizen and mentor. I tell my team that any good habits I have were Charis’ and what bad habits I have are entirely my own.

